# Betulinic Acid Exerts Cytoprotective Activity on Zika Virus-Infected Neural Progenitor Cells

**DOI:** 10.3389/fcimb.2020.558324

**Published:** 2020-11-05

**Authors:** Bruno R. R. Cavalcante, Luciana S. Aragão-França, Gabriela L. A. Sampaio, Carolina K. V. Nonaka, Moisés S. Oliveira, Gúbio S. Campos, Silvia I. Sardi, Beatriz R. S. Dias, Juliana P. B. Menezes, Vinícius P. C. Rocha, Erik A. Rossi, Bruno D. Paredes, Gabriele L. S. Martins, Kyan J. Allahdadi, Laisla R. Peixoto, José M. Barbosa-Filho, Bruno S. F. Souza, Milena B. P. Soares

**Affiliations:** ^1^ Center for Biotechnology and Cell Therapy, São Rafael Hospital, Salvador, Brazil; ^2^ D’Or Institute for Research and Education (IDOR), Rio de Janeiro, Brazil; ^3^ Gonçalo Moniz Institute, Oswaldo Cruz Foundation (FIOCRUZ), Salvador, Brazil; ^4^ Institute of Health Sciences, Federal University of Bahia, Salvador, Brazil; ^5^ Department of Pharmacy, Federal University of Paraíba, João Pessoa, Brazil

**Keywords:** Zika virus, neural progenitor cells, betulinic acid, apoptosis, neuroprotection

## Abstract

Zika virus (ZIKV), a member of the Flaviviridae family, was brought into the spotlight due to its widespread and increased pathogenicity, including Guillain-Barré syndrome and microcephaly. Neural progenitor cells (NPCs), which are multipotent cells capable of differentiating into the major neural phenotypes, are very susceptible to ZIKV infection. Given the complications of ZIKV infection and potential harm to public health, effective treatment options are urgently needed. Betulinic acid (BA), an abundant terpenoid of the lupane group, displays several biological activities, including neuroprotective effects. Here we demonstrate that Sox2^+^ NPCs, which are highly susceptible to ZIKV when compared to their neuronal counterparts, are protected against ZIKV-induced cell death when treated with BA. Similarly, the population of Sox2^+^ and Casp3^+^ NPCs found in ZIKV-infected cerebral organoids was significantly higher in the presence of BA than in untreated controls. Moreover, well-preserved structures were found in BA-treated organoids in contrast to ZIKV-infected controls. Bioinformatics analysis indicated Akt pathway activation by BA treatment. This was confirmed by phosphorylated Akt analysis, both in BA-treated NPCs and brain organoids, as shown by immunoblotting and immunofluorescence analyses, respectively. Taken together, these data suggest a neuroprotective role of BA in ZIKV-infected NPCs.

## Introduction

Zika virus (ZIKV) is a positive-sense single-stranded RNA arbovirus that belongs to the genus Flavivirus of the Flaviviridae family, which comprises other members that cause widespread morbidity worldwide (Huang et al., 2014), such as dengue virus (DENV), West Nile virus (WNV), yellow fever virus (YFV), and Japanese encephalitis virus (JEV) (Song et al., 2017). Although ZIKV infection had been thought to be a prominent disease, it has been underestimated for many years since the human infection was first described (MacNamara, 1954). Until the beginning of the epidemic in 2007, ZIKV was not as threatening to human health, since the infection was primarily considered to be mild. However, when ZIKV displayed an ability to compromise neurological systems, as seen by an escalating number of adults with Guillain-Barré syndrome and babies born with microcephaly (do Rosário et al., 2016), the World Health Organization declared it a public health emergency of international concern during the outbreak.

ZIKV has the ability to infect neural progenitor cells (NPCs), resulting in alterations in the expression of cell cycle-related proteins, induction of apoptosis and impaired production of new neurons (Li H. et al., 2016; Souza et al., 2016). In fact, Sox2^+^ NPCs population has been reported as highly affected by ZIKV (Souza et al., 2016). The proliferation and self-renewal of NPCs, as well as their differentiation, neuronal migration and maturation, are cardinal features for the regular developing embryonic mammalian brain (Li C. et al., 2016). Thus, the function of the neural circuitry can be drastically affected by variations in the number of neural cells produced during development, whether caused by disease or infection. Similarly, *in vitro* ZIKV infection of 3D cultures of human neurospheres compromised their growth and led to increased cell death (Garcez et al., 2016).

Despite several initiatives aimed at addressing greater knowledge on ZIKV biology, transmission, and pathogenesis of the disease and host’s response to infection, there are urgent needs that include the development of neutralizing molecules and anti-ZIKV agents, as there is no approved vaccine or specific therapy to prevent or treat ZIKV infection to date. Natural products play a key role in drug discovery as they exhibit a wide range of pharmacophores and favorable stereochemistry (Newman and Cragg, 2012). Terpenoids are one of the largest groups of natural products and their diversity of structures and functions have raised great interest in their commercial uses (Thoppil and Bishayee, 2011). Betulinic acid (BA) is a pentacyclic triterpenoid of the lupane group commonly found in the plant kingdom, and can be obtained from various plant species or from betulin, its metabolic precursor (Yogeeswari and Sriram, 2005). In this work betulinic acid had been re-isolated from *Zizyphus joazeiro* (Barbosa Filho et al., 1985). Several pentacyclic triterpenes display neuroprotective effects. As such, BA and its derivatives display a myriad of biologic effects (Amiri et al., 2020) which reports include anti-HIV (Baglin et al., 2003), antibacterial (Chandramu et al., 2003), and anti-helmintic actions (Enwerem et al., 2001), along with a robust cytotoxic activity against an extensive panel of tumor cell lines (Drag-Zalesinska et al., 2009; Chakraborty et al., 2015). Moreover, BA has been shown to possess some neuroprotective actions in brain lesions (Jiao et al., 2016) and neurological diseases (Navabi et al., 2018). Importantly, BA has been shown the ability to cross the blood brain barrier, making it a suitable molecule for the treatment of CNS disorders (Yogeeswari and Sriram, 2005).

In this work we aimed to evaluate the role of betulinic acid regarding its anti-ZIKV and neuroprotective activities in human neural progenitor cells, in both 2D and 3D cultures. Our results indicate a neuroprotective action of this natural compound in ZIKV and a possible involvement of the AKT pathway in BA protective activity.

## Materials and Methods

### Production of Betulinic Acid

Betulinic acid (BA) spectroscopically pure ≥ 98% was used in this study. It was isolated from the roots of Ziziphus joazeiro by a previously described method (Barbosa Filho et al., 1985). Betulinic acid spectrum analyses can be found in the supplementary material (**Figure S1**). The lyophilized compound had been resuspended in dimethyl sulfoxide (DMSO; Austin, TX, USA) and diluted in cell culture medium prior to the assays, reaching a final concentration of less than 0.1%, including negative controls.

### Cells and Viruses

The human induced pluripotent stem cells (iPSC) used in this study were generated using human cells in a procedure approved by the Ethics Committee of São Rafael Hospital (protocol number 19883113.0.0000.0048), as previously described (Martins et al., 2019). Participants read and signed the informed consent form of the study. Induced pluripotent stem cells (iPSC) were generated by reprogramming skin fibroblasts using episomal vectors, as previously described (Okita et al., 2011).

ZIKV (GenBank KU940228) was obtained from patient serum as previously described (Campos et al., 2015), and maintained in C6/36 cells, which are important for the replication of Flavivirus genus species. These cells were cultured at 28°C and 0% CO_2_ in Leibovitz L15 Medium (Thermo Fisher Scientific), supplemented with 5% fetal bovine serum (Thermo Fisher Scientific) and 10% phosphate tryptose broth (Sigma-Aldrich, St. Louis, MO, USA).

For ZIKV titration, VERO cells were plated in 96-well plates at the density of 1 x 10^4^ cells/well, 24 h prior to virus infection. After cell monolayer formation, viruses were thawed and a serial dilution (10^−1^ to 10^−8^) was performed on 10 replicates for each dilution. Fifty microliters of each dilution was placed in the respective wells and the cells were incubated at 37°C and 5% CO2 to promote adsorption and viral for 60–90 min. Next, 50 μl of 2% fetal bovine serum (FBS) Dulbecco’s modified Eagle medium (DMEM) medium was added in each well and the cells were taken to the incubator for five consecutive days to promote cytopathic effects, which were quantified for viral titer, according to Reed and Muench method (Reed and Munch, 1938). Titration of infectious virus obtained from the VERO cell culture yielded a value of 10^6^ TCID 50/ml.

### Induction of Neural and Neuronal Differentiation From Induced Pluripotent Stem Cells and Zika Virus Infection

Neural induction was performed in human monolayer iPSC culture, in Matrigel-coated plates (Corning, New York, USA), and incubated in STEMdiff Neural Induction Media, according to the manufacturer’s instructions. STEMdiff Neural Rosette Selection Reagent was used for isolation of neural rosettes and NPCs were maintained in STEMdiff Neural progenitor media (all from StemCell Technologies, Vancouver, Canada).

Neuronal differentiation was performed in NPCs monolayer cultures distributed in 24-well plates previously coated with poly-L-ornithin (15 μg/ml) and laminin (10 μg/ml) (both from Sigma-Aldrich) at a density of 5 x 10^4^ cells/cm^2^. STEMDiff Neuron Differentiation Medium (StemCell Technologies) was added to the culture for 7 days following STEMDiff neuronal maturation medium for 4 weeks according to the manufacturer’s instructions.

ZIKV infection was carried out on NPCs or neurons in 96-well plates (cell density of 10^4^ cells/well) or six-well plates (2.5 x 10^5^ cells/well) previously coated with Matrigel and incubated for 24 h at 37°C and 5% CO_2_. Culture medium was removed at the following day and the cells were incubated with ZIKV, diluted for the multiplicity of infection (MOI) of 1. Cells were incubated at 37°C and 5% CO_2_ for 90 min for ZIKV adsorption and penetration, with subsequent addition of neural progenitor medium for 48 h, with or without pharmacological treatment with different concentrations of betulinic acid (6.25, 12.5, 25, and 50 μM).

### Generation of Cerebral Organoids

Cerebral organoids were generated and cultured in a bioreactor as previously reported (Lancaster and Knoblich, 2014) with slight modifications. Briefly, iPSC were detached from Matrigel coating with 1 ml Accutase (StemCell Technologies) for 1 min, incubated at 37°C for 3–5 min. Wells were washed with fresh medium to collect undifferentiated iPSC and colonies were dissociated by up-and-down pipetting to generate a single-cell suspension. At day 0, embryoid bodies (EBs) were obtained by plating 9,000 cells/well on a U bottom low attachment 96 well plate (Corning) with DMEM/F12 media supplemented with 20% knockout serum replacement, 4 ng/ml bFGF, NEAA, glutamine, 100 µM B-mercaptoethanol, and 10 µM Rock inhibitor. After 6 days, EBs were transferred to new petri dishes containing neural medium (DMEM/F12, 1:100 N2 supplement, NEAA, glutamine, and 1 mg/ml heparin) until day 11, when organoids were transferred to Matrigel droplets and cultured in 1:1 DMEM/F12:neurobasal medium supplemented with 1:100 B27 without vitamin A, 1:200 N2, NEAA, insulin, beta-mercaptoethanol, and glutamine. Organoids were then transferred to stir flask bioreactors for long-term growth on day 15 in the same differentiation medium but with the use of regular B-27. Media was changed every 3 days.

For infection of cerebral organoids, each organoid (approximately 150,000 cells per 6-week organoid) was collected, inoculated with ZIKV stocks diluted in DMEM/F-12-based media (MOI of 1) and incubated for 1 h based on a previously described protocol with modifications (Watanabe et al., 2017). Thereafter, infected organoids were maintained in a medium consisting of fresh culture medium supplemented with 10 µM BA, which was changed every 2–3 days for 7 days after infection.

### Anti-Zika Virus Activity on Neural Progenitor Cells

After infection with ZIKV, BA was added to NPCs cultures, in order to determine the concentration that inhibits viral replication (IC_50_) or cell viability (CC_50_) by 50%. The IC_50_ and CC_50_ values were determined based on the percent inhibition of viral infection and cell viability of the negative control, i.e., without BA. In all experiments, five different drug concentrations were considered. The non-linear regression calculation to obtain the IC_50_ and CC_50_ values was performed on GraphPad Prism 8 software (GraphPad Prism 8.2.0, La Jolla, CA, USA). Chloroquine was used as positive control (Delvecchio et al., 2016).

### Cytotoxicity Evaluation

Determination of lethal concentration to 50% of cell population (CC_50_) was performed by alamarBlue Assay (Invitrogen, Carlsbad, CA, USA), following the manufacturer’s instructions. NPCs were cultured in 96-well plates (10^4^ cells/well) and incubated at 37°C and 5% CO_2_ for 24 h. Next, BA was added at different concentrations and the plates were incubated for 48 h. After incubation, the wells were washed with saline, and then 10% alamarBlue^®^ reagent (Invitrogen) was added to the culture medium. The cells were incubated for an additional 18 h, and then the colorimetric readings of the 96-well plate with the wavelengths of 570 and 600 nm were performed. The calculation for obtaining the CC_50_ value was performed using the non-linear regression on GraphPad Prism 8 software.

### Evaluation of Neuroprotective Activity

Determination of half maximal effective concentration (EC_50_) was performed as follows: NPCs were cultured in 96-well culture plates (10^4^ cells/well) and incubated at 37°C and 5% CO_2_ for 24 h. Then, BA was added at different concentrations (0.78, 1.56, 3.10, 6.25, 12.5, 25, and 50 μM) and the plates were incubated for 48 h. Following incubation time, the wells were fixed with 4% paraformaldehyde (Electron Microscopy Sciences, Hatfield, PA, USA) for 15 min for image analysis on Operetta High Content Screening System (Perkin Elmer, Waltham, MA, USA) and Software Harmony 3.5.2 (Perkin Elmer). For the analysis, nine fields were photographed for segmentation and identification of the host cell quantification. Cell targeting was performed using Harmony Digital Phase Imaging algorithms.

The EC_50_ value for cytoprotection were determined based on the percent stimulus of BA treatment following viral infection and cell viability of the positive control, i.e., cultures in absence of drugs in ZIKV-infected NPCs. In all experiments, seven drug concentrations were considered. The sigmoidal concentration-response calculation to obtain the EC_50_ values was performed using the GraphPad Prism 8 software (GraphPad Software).

### Western Blot Analysis for Akt Signaling Pathway

Western blot analysis was performed as previously detailed (Jiao et al., 2016). Succinctly, total protein of the NPCs pool of 3 x10^6^ cells homogenate cultured in six-well plate was collected and 100 µg of protein samples was separated by sodium dodecyl sulfate-polyacrylamide gel electrophoresis (SDS–PAGE), following transference to nitrocellulose membrane. The membrane was blocked with 5% powdered milk in PBS 1X pH 7.4 with 0.1% Tween 20 for 1 h at room temperature. Membranes were then incubated with the primary antibodies (human/mouse/rat Akt pan specific antibody, 1:2500, R&D Systems; human/mouse/rat Phospho Akt (S473) pan specific antibody, 1:400, R&D Systems; mouse polyclonal antibody against actin, 1:5,000 (Abcam, Cambridge, UK) overnight at 4°C. Blots were washed three times in PBS/Tween 20 (0.1%) with 3% powdered milk and then incubated with a horseradish peroxidase-conjugated secondary antibody in 3% powdered milk and 0.1% Tween 20 in PBS for 1 h at room temperature. After washing, blots were developed using an enhanced horseradish peroxidase/luminol chemiluminescence reagent (Abcam) according to the manufacturer’s protocol. Quantitative analysis was performed using a Luminescent Image Analyzer and ImageQuant Las 4000 software, with the ImageJ software (NIH Image, USA). Actin was used as normalizing control.

### Immunofluorescence Analyses

NPCs cultured in glass coverslips were fixed with 4% paraformaldehyde (Electron Microscopy Sciences, Hatfield, PA, USA) for 15 min, permeabilized with 0.1% Triton X-100 (Sigma-Aldrich) for 15 min and blocked with 5% bovine serum albumin (BSA/PBS) (Sigma-Aldrich) for 1 h. After incubation time, the following primary antibodies diluted in 1% BSA were used: cleaved anti-caspase 3 (1:200, Cell Signaling Technology, Danvers, MA, USA), anti-DCX, (1:300, Santa Cruz Biotechnology, Dallas, TX, USA), Anti-Nestin (1:200; Millipore, Billerica, MA, USA), anti-Sox2 (1:500, Cell Signaling Technology). For ZIKV labeling, the primary monoclonal antibody against Flavivirus E protein (MIAF, obtained from WRECVA, diluted 1:1,000), was kindly provided by Dr. Nikos Vasilakis (University of Texas Medical Branch). All antibodies were incubated overnight at 4°C. The following secondary antibodies were used (diluted 1:1,000): Alexa Fluor 488 or 568 IgG anti-mouse, Alexa Fluor 568 IgG anti-rabbit, Alexa Fluor 488 IgG anti-mouse, Alexa Fluor 488 IgG anti-mouse, anti-human IgG Alexa Fluor 488 (all Thermo Fisher Scientific). The coverslips were subjected to successive washes with 0.05% Tween 20 diluted in PBS. Nuclear staining was performed with DAPI (Vector Laboratories, Burlingame, CA, USA). The images were captured on the A1+ confocal microscope (Nikon, Tokyo, Japan) or the FluoView 1000 confocal microscope (Olympus, Tokyo, Japan).

For image analysis on Operetta High Content Screening System (Perkin Elmer, Waltham, MA, USA) and Software Harmony 3.5.2 (Perkin Elmer), nine fields/well of labeled samples were photographed for segmentation and identification of the host cell and infection quantification. Cell targeting was performed using harmony software algorithms. First, the nuclei were detected as objects in the channel Hoechst33342 with area smaller than 30 μm^2^ and contrast smaller than 0.05. Quantification for NPCs characterization was performed based on the ratio as follows: correspondent NPCs staining area (i.e., NESTIN, SOX2, and PAX6): Hoechst33342 staining area.

Infected cells were then selected based on the median fluorescence intensity of Alexa Fluor 488, located in the cytoplasm of the cell (median > 100). Based on the analysis of the images using these parameters, the number of infected cells per well was quantified. The infection inhibition of BA was determined on the basis of the number of infected and treated cells (X test) relative to the number of infected and untreated cells (Y control), according to the equation below. A control of uninfected cells was used to normalize the fluorescence intensity.

$$Infection\;inhibition\;\%=\left(\frac{X\;test}{Y\;control}x\;100\right)-100$$

Neurotoxicity was evaluated on High Content Screening (Operetta) system by counting the number of NPCs previously labeled with Hoechst33342 (Thermo Fisher Scientific), a nuclear marker, after being infected with ZIKV followed by treatment with BA and incubation for 48 h. The analysis was performed on Harmony Software 3.5.2 (Perkin Elmer). Initially, the core was detected as objects in the Hoechst33342 channel with area smaller than 30 μm^2^ and contrast smaller than 0.05. Based on the analysis of the images using these parameters, the number of infected cells/well was quantified.

### Gene Expression Analysis

NPCs were seeded in six-well plates (3 x 10^5^ cells/well) and cultured for 48 h. Culture medium was removed from three wells and the cells were washed with 1 ml of ice-cold PBS and centrifuged at 350 x g for 5 min at 4°C. The supernatant was discarded and the pellet used for RNA extraction.

Total RNA was extracted with TRIzol^®^ (Thermo Fisher Scientific) following manufacturer’s protocol. RNA pellets were resuspended in 10 μl of RNase-free water and stored at −80°C. For complementary DNA synthesis (cDNA), 1 μg of RNA per sample was used in the high-throughput cDNA reverse transcription SuperScript VILO cDNA Synthesis Kit (Thermo Fisher Scientific), following manufacturer’s instructions. To quantify the expression of the *SOX2*, *MAP2*, and *TUJ1* genes, 60 ng of cDNA template was used; 5 μl of TaqMan Master Mix 2x (Thermo Fisher Scientific) and 0.5 μl of probes in the 20x concentration, for a final volume of 10 μl (all from Integrated DNA Technologies, Coralville, Iowa, EUA). All reactions were performed in triplicate on ABI7500 thermal cycler (Thermo Fisher Scientific) under standard thermal cycling conditions. Mean Ct values (cycle limit) were used to calculate the expression of target gene, and normalized with housekeeping genes (*GUSB*, hs99999908_m1, *HPRT* hs99999909_m1; *GAPDH*, hs99999905_m1). All experiments were performed under DNAse/RNAse free conditions. The results were analyzed through 2^−ΔCt^ method (Schmittgen and Livak, 2008). Graphs were generated using the Prism 6 GraphPad program (GraphPad Software). All oligonucleotide primers used in this study are shown in **Table S1**. Amplification of the *AXL* and *DCX* genes were obtained by TaqMan probes (hs01064444_m1and hs00167057_m1, respectively).

### Caspase Enzymatic Activity Test

NPCs were seeded in 96-well plates (10^4^ cells/well) and incubated at 37°C and 5% CO_2_. After 24 h incubation, ZIKV infection at a MOI of 1 was performed, followed by treatment with BA at concentrations of 6.25, 12.5, 25, and 50 μM and incubated for 48 h. The enzymatic activity of 3/7 caspases was measured using a Caspase-Glo Assay Kit, according to the manufacturer’s protocol (Promega, Madison, WI, USA). In brief, 100 μl of Caspase-Glo reagent was added to the cells and taken to a plate shaker at 100 rpm and incubated at room temperature for 90 min. The luminescence of each sample was measured on a Glomax 20/20 Luminometer (Promega).

### Target Genes Related to Betulinic Acid

STITCH Database (http://stitch.embl.de/, v. 5.0) with the “*Homo sapiens*” species setting was used for identification of 10 target genes linked to betulinic acid and a confidence score of > 0.7 (Kuhn et al., 2008; Szklarczyk et al., 2016). UniProt (http://www.uniprot.org/) was utilized to retrieve gene information, including name, gene ID and organism.

### Statistical Analysis

Statistical analysis of ZIKV-infected NPCs was performed by GraphPad Prism 8 software using non-linear regression tests, one-way ANOVA, two-way ANOVA, and unpaired Student’s *t*-test. When necessary, Tukey or Newman–Keuls post-test was used and values of *p <*0.05 were considered statistically significant.

## Results

### Generation and Characterization of Human Induced Pluripotent Stem Cells-Derived Neural Progenitor Cells

**Figure 1:**
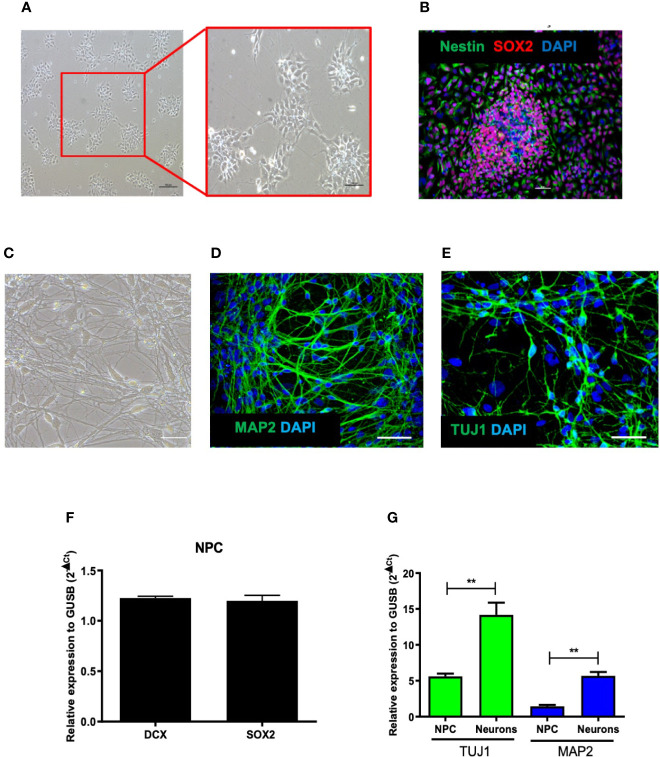
Characterization of human-derived neural progenitor cells (NPCs). **(A)** Culture of NPCs observed by phase contrast microscopy after differentiation of human induced pluripotent stem cells (hiPSC). **(B)** Confocal microscopy of cultures of NPCs co-stained with Nestin, Sox2, and DAPI. **(C)** Culture of neurons observed by phase contrast microscopy after NPCs differentiation. **(D, E)** Confocal microscopy of cultures of NPCs-derived neurons labeled with MAP2, TUJI, and DAPI. **(F)** Gene expression of NPCs markers: DCX and SOX2. **(G)** Gene expression of TUJ1 and MAP2 in NPCs and neuron cultures. Bar scale = 100 μm. Expression related to GUSB. **p < 0.005.

Induced pluripotent stem cells (iPSC) were differentiated into NPCs and characterized by expression of the cell markers nestin and Sox2 (**Figures 1A, B**). The NPCs were further induced to differentiate into neurons for 1 month, which displayed cellular clusters, extensive neurite proliferation and branching, in addition to being positive for the neuronal differentiation markers MAP2, DCX, and TUJ1 (**Figures 1C–E**). Both cultures, NPCs and neurons, were evaluated for the expression profile of genes relevant for their differentiation processes. NPCs cultures showed the expression of DCX and SOX2, as well as markers of neuronal commitment TUJI and MAP2, the latter being more expressed in neurons (**Figures 1F, G**). Quantification of stained NPCs by high content imaging analysis revealed a population comprising Nestin^+^ (99.78 ± 0.2%), Sox2^+^ (77 ± 11%), Pax6^+^ (59.41 ± 10%) cells.

### Zika Virus Infection on Neural Progenitor Cells and Neural Progenitor Cell-Derived Neurons

**Figure 2:**
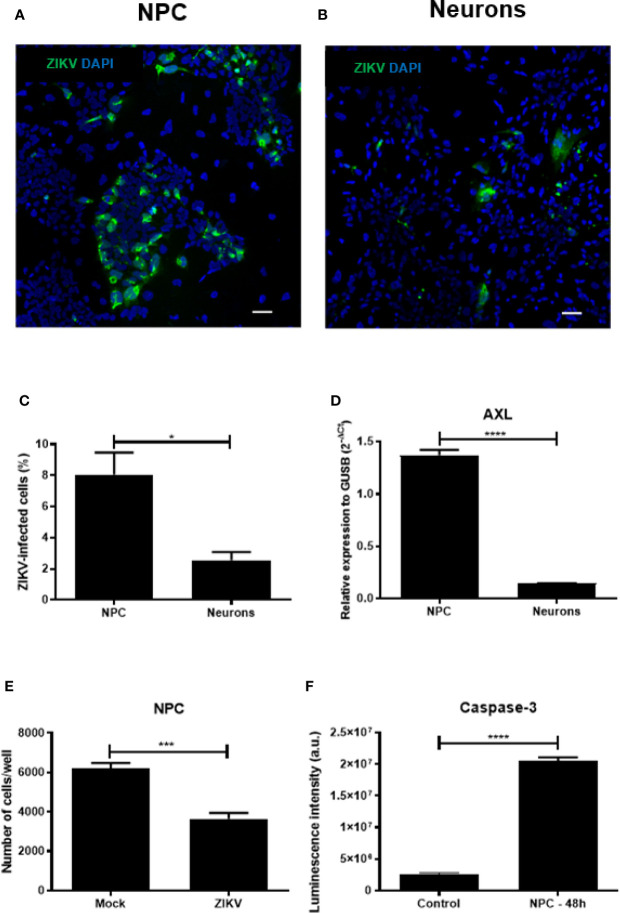
Zika virus (ZIKV) infection in human neural progenitor cells (NPCs) and neurons. **(A)** Representative images of NPCs and neurons after 48 h of ZIKV infection. **(B)** Quantification of ZIKV-infected cell types. **(C)** Number of NPCs and neurons after 48 h of ZIKV infection. **(D)** Gene expression of AXL receptor in NPCs and neurons. **(E)** Number of NPCs after 48 h of ZIKV infection. **(F)** Determination of cleaved caspase-3 in NPCs culture after 48 h of ZIKV infection. Multiplicity of infection (MOI) of 1. Bar scale = 100 μm. Expression related to GUSB. a.u. = arbitrary units. *p < 0.05, ***p < 0.001, ****p < 0.0001.

We first compared the susceptibility of NPCs and neurons after ZIKV infection. A higher percentage of infected cells was found in NPCs compared to their neuronal counterparts after 48 h of infection, as shown by quantification of ZIKV^+^ cells (**Figures 2A–C**). Next, we compared the expression of AXL, shown to be one of the receptors for ZIKV entrance (Nowakowski et al., 2016), in the two cell cultures. The comparison of NPCs and neurons showed a higher expression of AXL receptor by gene expression analysis in NPCs when compared to neurons (**Figure 2D**).

Moreover, the cell density of NPCs cultures decreased after ZIKV infection (**Figure 2E**). Next, we confirmed the susceptibility of NPCs to ZIKV infection by evaluating the caspase 3 enzymatic activity, indicative of apoptotic process. As presented in **Figure 2F**, caspase 3 enzymatic activity was markedly increased in NPCs 48 h after ZIKV infection. These results confirmed that NPCs are strikingly susceptible to ZIKV infection by apoptotic cell death.

### Effects of Betulinic Acid on Zika Virus-Infected Neural Progenitor Cells

**Figure 3:**
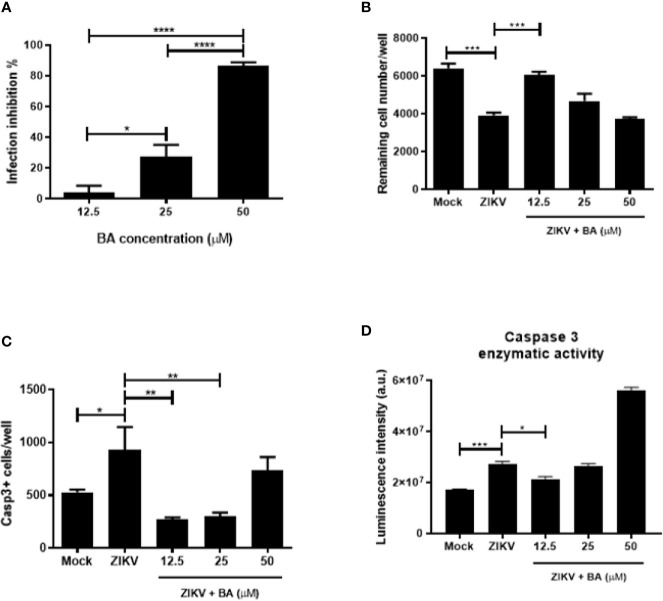
Betulinic acid treatment in Zika virus (ZIKV)-infected neural progenitor cell (NPCs). **(A)** Percentage of infection inhibition after treatment with betulinic acid at the concentrations of 50, 25, and 12.5 μM. **(B)** Number of remaining cells after ZIKV infection following betulinic acid (BA) treatment. **(C)** Number of Caspase 3^+^ cells after ZIKV infection following BA treatment. **(D)** Detection of enzymatic activity of cleaved caspase 3 by luminescence. Multiplicity of infection (MOI) of 1. *p < 0.05, **p < 0.005, ***p < 0.001, and ****p < 0.0001. Values represent the mean ± SEM of three replicates. a.u. = arbitrary units.

To investigate the effects of BA, we first evaluated its cytotoxicity to NPCs. BA induced low toxicity in uninfected NPCs, with a CC_50_>50 µM. When tested in ZIKV-infected NPCs cultures, BA showed a concentration-dependent antiviral activity (**Figure 3A**), with an IC_50_ value of 20.30 ± 1.50 µM. The highest BA concentration tested (50 µM) resulted in a viral inhibition effect close to 100% (**Figure 3A**). However, when the cell number was evaluated 48 h after infection, we observed a significant reduction in NPCs numbers in cultures treated with 50 µM BA (**Figure 3B**). In contrast, the lowest BA concentration tested (12.5 µM), while displaying a mild antiviral potential, maintained the number of remaining cells after ZIKV infection similar to mock-infected NPCs (**Figure 3B**; **Figure S2**). Chloroquine at the concentrations tested did not prevent NPCs loss due to ZIKV infection (data not shown).

To further corroborate the fact that lower concentrations of BA protect NPCs cultures from ZIKV-induced cell death, we evaluated the expression of cleaved caspase 3 after BA treatment in NPCs after 48 h of ZIKV infection. When BA was tested at 12.5 μM, lower numbers of Casp3^+^ NPCs were found, whereas in concentrations higher than 25 μM, we found an increase of cleaved caspase 3 and low cell viability (**Figure 3C**). The same pattern could be observed by caspase 3 enzymatic activity using a luminescence assay (**Figure 3D**). Taken together, these data suggest that BA prevented ZIKV-induced NPCs death by apoptosis at 12.5 μM.

### Effects of Betulinic Acid in Cerebral Organoids

**Figure 4:**
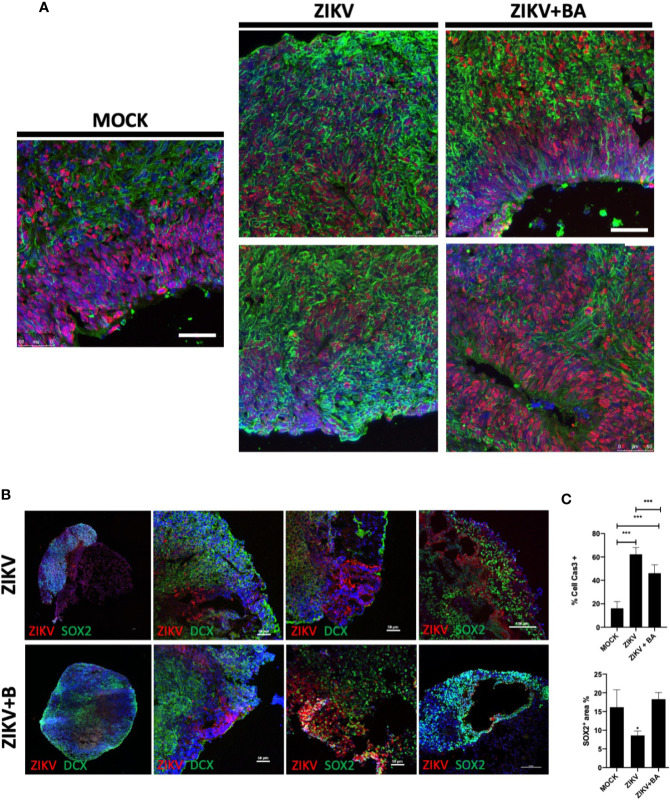
Betulinic acid treatment in Zika virus (ZIKV)-infected 6-week cerebral organoids. **(A)** Representative confocal microscopy images of histological sections obtained from cerebral organoids non-infected (MOCK), ZIKV-infected (ZIKV), or ZIKV-infected treated with betulinic acid at the concentration of 10 μM (ZIKV+BA) stained for SOX2 (red) and TUJ1 (green) neural progenitor cell (NPCs) and neuron markers, respectively, and nuclei counterstained with DAPI (blue). Scale bars = 50 µm**. (B)** Representative images of the organoids stained for ZIKV (red) and either SOX2 or DCX (green), as indicated. Scale bars = 50 µm**. (C)** Quantification of Casp3+ and SOX2^+^ cells in the cerebral organoids area after treatment with betulinic acid. Multiplicity of infection (MOI) of 1. *p < 0.05, ***p < 0.001. Values represent the mean ± SEM of three replicates.

NPCs play a crucial role in the developing brain, and previous studies have revealed that ZIKV can affect brain organoid development *in vitro* (Garcez et al., 2016; Janssens et al., 2018). To further examine the protective action of BA in Sox2^+^-NPCs population, we generated cerebral organoids from iPSCs and performed ZIKV infection (**Figures 4A, B**). ZIKV-infected organoids presented a marked reduction in the Sox2^+^-NPCs and increase in Casp3^+^ cells, compared to uninfected (mock) organoids (**Figure 4C**). In BA-treated organoids, however, the Sox2^+^-NPCs area was increased, while Casp3^+^ cells were decreased, when compared to ZIKV-infected organoids (**Figure 4C**). Additionally, ZIKV caused marked alterations in the morphology and structure of brain organoids, such as irregular borders, presence of cavities, disruption of ventricular zone-like areas and lower cortical-committed DCX^+^ cells in the outer layer of organoids, which was found to be more preserved in the organoids treated with BA (**Figures 4A, B**).

### Activation of Akt Signaling Pathway by Betulinic Acid in Zika Virus Infected Cultures

**Figure 5:**
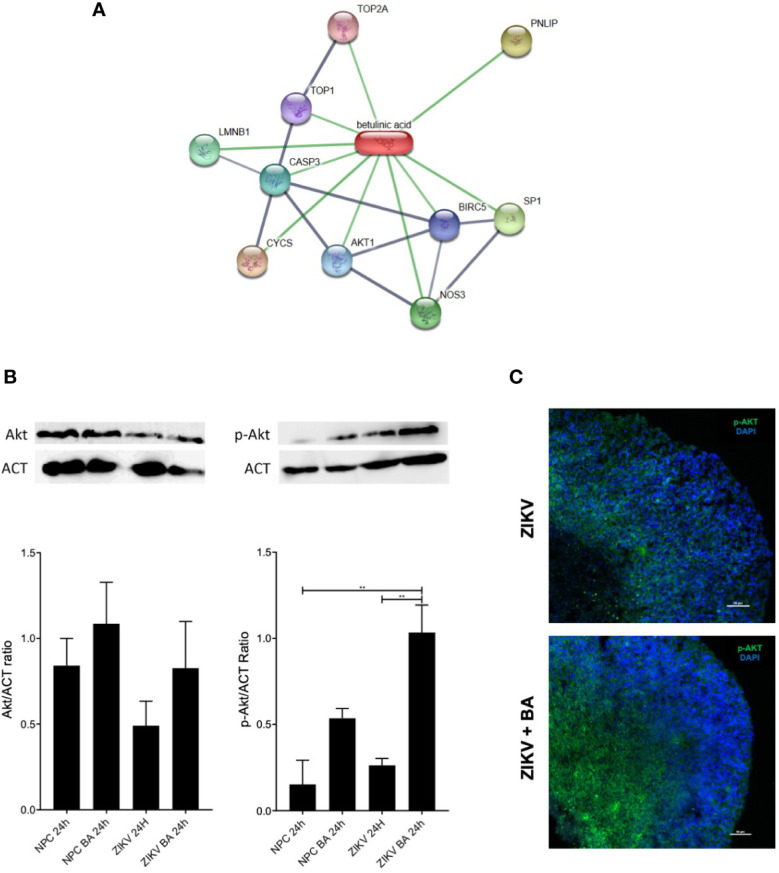
Betulinic acid displays neuroprotective effect on neural progenitor cell (NPCs) population through activation of AKT signaling pathway. **(A)** Gene interaction map overlapped with betulinic acid via the STITCH database. Stronger associations are represented by thicker lines. Protein-protein interactions are shown in grey and chemical-protein interactions in green. **(B)** Protein expression levels of AKT and phosphorylated AKT in ZIKV-infected NPCs pool after 48-h treatment with betulinic acid at the concentration of 10 μM. **(C)** Representative confocal microscopy images of BA-treated brain organoids incubated for 7 dpi at the concentration of 10 μM stained for AKT and phosphorylated AKT (p-AKT). Multiplicity of infection (MOI) of 1. **p < 0.005. Values represent the mean ± SEM of three replicates. MOI of 1.

Lastly, we sought to better understand the mechanism by which BA exerts its neuroprotective action by using the chemical and protein interactions database STITCH. The genes CASP3, AKT1, BIRC5, TOP1, TOP2A, NOS3, SP1, PNLIP, LMNB1, and CYCS were the most enriched by BA in this network (**Figure 5A**). The identified targets were mainly associated with regulation of apoptotic processes, cell proliferation, and to mitotic cell cycle.

Akt pathway was selected to be validated, as it has been previously reported that ZIKV displayed an important role in deregulating Akt-mTOR signaling pathway in human neural stem cells (Liang et al., 2016). To investigate if the phosphorylation dynamics was altered following BA treatment in ZIKV-infected cultures, we performed western blotting analysis on lysates of NPCs cultures, using anti-Akt and anti-phosphorylated Akt antibodies (**Figure 5B**). While no differences were observed when total Akt antibodies were tested, anti-pAkt antibodies showed an increased phosphorylation of Akt in ZIKV infected cultures after BA treatment, compared to untreated controls (**Figure 5B**). Moreover, we found an augmented expression of phosphorylated Akt in BA treated, ZIKV-infected brain organoids compared to ZIKV-infected controls by immunofluorescence analysis (**Figure 5C**).

## Discussion

Considering the need to identify new drugs with antiviral activity and the fact that there is no ZIKV-specific antiviral therapy, in this work we sought to investigate the effects of BA, a natural compound with previously described neuroprotective activities, in ZIKV-infected human NPCs cultures as a disease model. By using a high content screening assay based on ZIKV-infected NPCs, we could determine the concentration-dependent anti-ZIKV and neuroprotective actions of BA. Moreover, we validated the neuroprotective action of BA in 3D cultures using NPCs-derived brain organoids.

NPCs are multipotent cells capable of differentiating into the major neural phenotypes, shown to be permissive to ZIKV infection and very susceptible to the effects of viral replication (Ferraris et al., 2019), thus shedding light on the developmental abnormalities perceived in gestational ZIKV infections. The paucity of biologically relevant screening models hampers the discovery of consistent treatments for ZIKV infection. Therefore, promoting a drug screening assay with human NPCs allows the neurotoxicity assessment of drug candidates, as it can predict risks related to chemical exposure in humans through a cellular model which is associated with the effects of ZIKV on neurological development. This was shown here by the loss of Sox2^+^-NPCs, causing a reduction in cell number due to apoptotic cell death, confirming previous results from our group (6). This is in accordance with the work by Dang and colleagues, showing that ZIKV infection decreases the population of neural progenitors in human brain organoids derived from human embryonic stem cells (hESC) (Dang et al., 2016). In fact, growth arrest and cell death have been shown to be induced by different strains of ZIKV in early differentiating neuroprogenitor cells, even at a very low multiplicity of infection (Devhare et al., 2017). The susceptibility of NPCs to ZIKV is dependent on the stage of the cultures, being the less differentiated more susceptible to ZIKV-induced cell death (Ferraris et al., 2019). Furthermore, it has already been noted that ZIKV-infected Sox2^+^-NPCs are induced to abnormalities in mitosis and lead to apoptotic cell death (Souza et al., 2016). Taken together, these results indicate an impaired proliferation observed in NPCs which progresses to cell death by apoptosis.

When we compared NPCs to neurons upon infection, we found a higher percentage of ZIKV-infected NPCs than neurons. This finding correlated with a higher expression of AXL on NPCs. As demonstrated by Hamel *et al*., ZIKV enters the cell through the phosphatidylserine receptor AXL and positively regulates the autophagy pathway, leading to increased viral replication in autophagosomes as an infection reservoir (Hamel et al., 2015). Although AXL appears to be not so expressed in mature neurons (Nowakowski et al., 2016), this receptor is not the only receptor for ZIKV infection. Other additional receptors, which have not been included in this work, such as members of the TIM and TAM receptor families (Hamel et al., 2015), may be important for viral entry.

By testing both the antiviral and neuroprotective activities of BA, we found here that this natural compound exerts dual effects, depending on the concentration tested. The fact that BA at the maximum concentration tested (50 µM) is not toxic to uninfected NPCs but promoted a significant cell loss in ZIKV-infected cultures, suggests a cytotoxic interaction between BA and ZIKV-induced factors. In this regard, synergistic cytotoxicity from drugs and cytokines have been previously described (Maiuri et al., 2017; Olmo et al., 2017). Indeed, it has been demonstrated that ZIKV infection is capable of triggering the production of proinflammatory cytokines, such as TNF and IL-β, and glutamate, mediators shown to induce neuronal cell death in ZIKV (Olmo et al., 2017). Moreover, apoptosis-induced microcephaly alterations have been linked to high proinflammatory cytokines production, for instance TNFα and IL-1β (de Sousa et al., 2018). In our study, when ZIKV-infected NPCs were treated BA after infection, the lower concentration of BA had a milder antiviral activity but caused less toxicity to the NPCs, protecting these cells from ZIKV-induced death. Additionally, the Sox2^+^-NPCs population found in BA-treated brain organoids was significantly increased when compared to ZIKV-infected controls, and presented with more well-preserved structures. Taken together, the results suggest that BA, at a lower concentration, had a neuroprotective role independent of an antiviral activity.

Chemical modification of BA structure may lead to the production of derivatives with improved desired effects. This approach has already been used in order to reduce its cytotoxicity and to achieve a better solubility and anti-*Trypanosoma cruzi* activity (Meira et al., 2016). Furthermore, a possible association is a drug combination therapy with another anti-ZIKV drug can be considered, such as mefloquine (Barrows et al., 2016) and chloroquine (Delvecchio et al., 2016), already screened in other assays. Thus, combining the effects that prevent viral replication with reduced cell death of NPCs may confer a double benefit.

An in-silico approach was attempted in this work to identify potential molecular targets of BA involved in cell protection in ZIKV-infected NPCs. The Akt signaling pathway displays relevant roles in regulating various cell functions, such as metabolism, cell growth, apoptosis and survival, in response to extracellular stimuli (Song et al., 2005). In fact, by performing proteomics analysis, a recent report has shown a strong downregulation of the AKT–mTOR signaling pathway in NPCs (Scaturro et al., 2018). BA was capable of activating/PI3K/Akt signaling pathway in hippocampal neurons in a neuronal injury model induced by oxygen and glucose deprivation/reperfusion (OGD/R), promoting neuronal survival after cerebral ischemia/reperfusion injury (Jiao et al., 2016). Here we report that betulinic acid activates the AKT cell survival pathway in ZIKV cultures, suggesting a role of this signaling pathway in BA’s protective role in cell survival upon ZIKV infection.

Based on our data, it is not possible to affirm that BA-induced actions are specific in the context of ZIKV infection, and there are other reports of antiviral—including against flaviviruses—and neuroprotection in the scientific literature. Several works have shown that pentacyclic triterpenes display neuroprotective outcomes. Maslinic acid found in *Olea europaea* species, for instance, induces synaptogenesis and axonal regeneration in cerebral ischemia model though the regulation of Akt/GSK-3β signaling pathway (Qian et al., 2015). Similarly, in cerebral ischemia models, asiatic acid isolated from *Centella asiatica* reduces glutamate-induced cognitive deficits, blood-brain barrier permeability, and mitochondrial injury (Krishnamurthy et al., 2009; Xu et al., 2012). Lastly, ursolic acid from the *Oleaceae* family shows neuroprotective actions through the activation of Nrf2 pathway in a cerebral ischemia mouse model (Li et al., 2013). To date, the neuroprotective role conferred by BA has neither been investigated in NPCs derived from human-derived iPSC, nor the relevance of this role when considering ZIKV infection.

## Conclusion

Our work provides new insights into the role of BA regarding neurotoxicity on ZIKV-infected NPCs, as well as its neuroprotective role, which may lead to a novel approach for therapeutic intervention. Our study suggests that BA is a drug candidate as a neuroprotective agent in ZIKV-infected NPCs, making them less susceptible to the virus-induced cell death.

## Data Availability Statement

The raw data supporting the conclusions of this article will be made available by the authors, without undue reservation.

## Ethics Statement

The studies involving human participants were reviewed and approved by Ethics Committee of São Rafael Hospital (approval number 19883113.0.0000.0048). The patients/participants provided their written informed consent to participate in this study.

## Author Contributions

BS and MS conceived the study and designed the experiments. GC and SS isolated, expanded, and tittered ZIKV. LP and JF carried out experiments to obtain betulinic acid. BC, LA-F and GM performed the experiments to generate NPCs and neurons and promoted ZIKV infection and betulinic acid treatment. GAS and MO conducted experiments to generate brain organoids. BC, ER, VR, BP, and KA performed immunofluorescence and analyzed the data. LA-F, BD and JM conducted western blot analysis. BC and CN performed RNA isolation and gene expression analyses. BC, LA-F, CN, GC, BS, and MS participated in data discussion, writing, and editing the manuscript. All authors contributed to the article and approved the submitted version.

## Funding

This research was funded by the Brazilian National Council for Scientific and Technological Development (CNPq), DECIT/MS (grant number 443909/2018-0), INCT-REGENERA (grant number 465656/2014‐5) and the Coordination for the Improvement of Higher Education Personnel (CAPES/Zika Fast track).

## Conflict of Interest

The authors declare that the research was conducted in the absence of any commercial or financial relationships that could be construed as a potential conflict of interest.
