# Supplementary figures and images for: Betulinic Acid Exerts Cytoprotective Activity on Zika Virus-Infected Neural Progenitor Cells
Source: Front Cell Infect Microbiol. 2020 Nov 5;10:558324. doi: 10.3389/fcimb.2020.558324 (PMC7674920; doi:10.3389/fcimb.2020.558324)

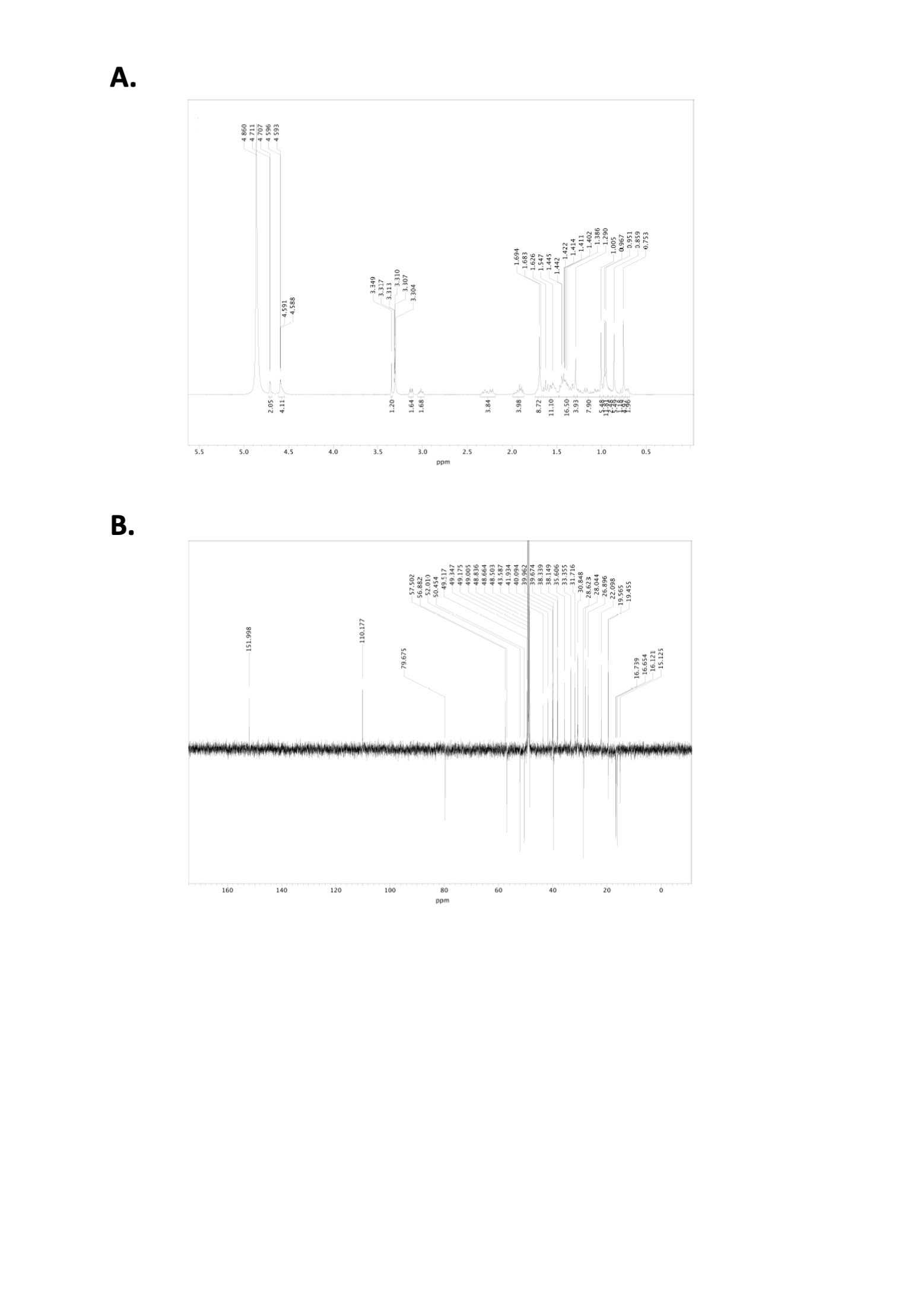

Supplement: Supplementary file 1 [file Image_1.jpeg]

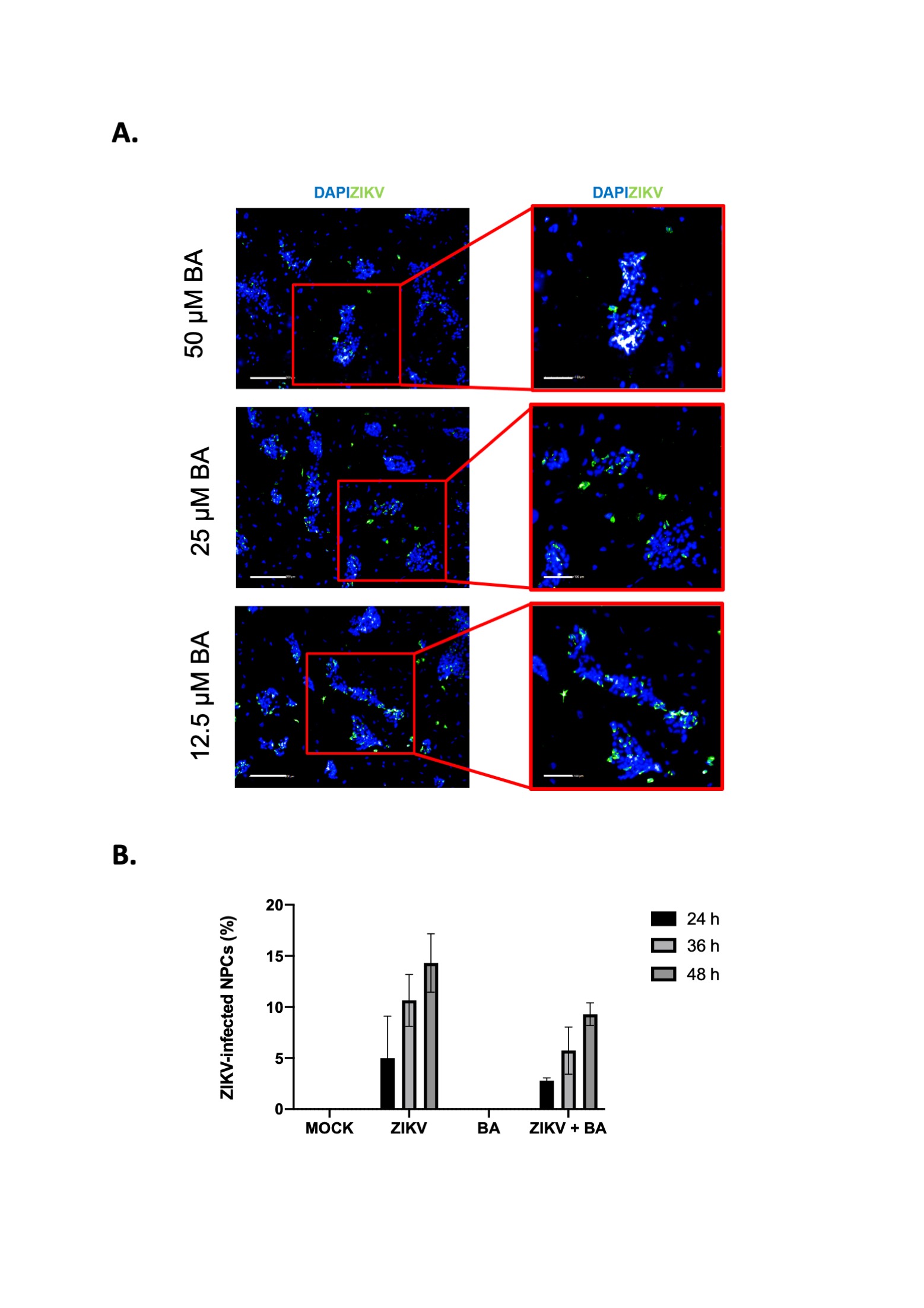

Supplement: Supplementary file 2 [file Image_2.jpeg]
