# Supplementary material for: Betulinic Acid Exerts Cytoprotective Activity on Zika Virus-Infected Neural Progenitor Cells
Source: Front Cell Infect Microbiol. 2020 Nov 5;10:558324. doi: 10.3389/fcimb.2020.558324 (PMC7674920; doi:10.3389/fcimb.2020.558324)
Supplement: Supplementary file 3 [file Table_1.docx]

**SUPPLEMENTARY MATERIAL**

**SUPPLEMENTAL FIGURE LEGENDS**

**Figure S1. Betulinic acid spectrum analyses.** 1H (A) and 13C-APT NMR (B) spectra of betulinic acid.

**Figure S2. Betulinic acid treatment in ZIKV-infected NPC. (A)** Representative confocal microscopy images of BA-treated NPCs incubated for 48 h after ZIKV infection at the concentrations of 12.5, 25, and 50 μM and MOI of 1. (B) Infection kinetics evaluated by High content imaging of ZIKV+ cells, comparing Mock and ZIKV-infected NPCs with 12.5 μM BA-treated NPCs either non-infected (BA) or ZIKV-infected (ZIKV+BA).

**SUPPLEMNETAL TABLE**

**Table S1**. Primer sets for PCR used in this study.

| **Primer** | **Forward sequence** | **Reverse sequence** |
| --- | --- | --- |
| MAP2 | 5'-CTTCAGCTTGTCTCTAACCGAG-3' | 5'-CTGCAACTATTCAAGGAAGTGG-3' |
| SOX2 | 5'-CACACTGCCCCTCTCAC-3' | 5'-TCCATGCTGTTTCTTACTCTCC-3' |
| TUJ1 | 5'-TTTGGACATCTCTTCAGGCC-3' | 5'-TTTCACACTCCTTCCGCAC-3' |
